# Beyond IC_50_*s*: Towards Robust Statistical Methods for *in vitro* Association Studies

**DOI:** 10.4172/2153-0645.1000121

**Published:** 2014-03-01

**Authors:** Andrew Beam, Alison Motsinger-Reif

**Affiliations:** 1 Bioinformatics Research Center, North Carolina State University, Raleigh NC, USA; 2 Department of Statistics, North Carolina State University, Raleigh NC, USA

## Abstract

Cell line cytotoxicity assays have become increasingly popular approaches for genetic and genomic studies of differential cytotoxic response. There are an increasing number of success stories, but relatively little evaluation of the statistical approaches used in such studies. In the vast majority of these studies, concentration response is summarized using curve-fitting approaches, and then summary measure(s) are used as the phenotype in subsequent genetic association studies. The curve is usually summarized by a single parameter such as the curve’s inflection point (e.g. the EC/IC_50_). Such modeling makes major assumptions and has statistical limitations that should be considered. In the current review, we discuss the limitations of the EC/IC_50_ as a phenotype in association studies, and highlight some potential limitations with a simulation experiment. Finally, we discuss some alternative analysis approaches that have been shown to be more robust.

## Discussion

Association mapping (with either DNA level variation or gene expression data) in pharmacogenomics has been impeded by the reliance on clinical trials for samples.

Genetic studies nested within clinical trials face the limited ability to enroll enough human subjects, ethical constraints, and the presence of uncontrolled confounders all of which may limit the capability to identify loci involved in drug-response [1]. To address these limitations, *in vitro* association studies have been proposed as an alternative to human-based studies because they address many of these concerns for certain types of drugs [1,2]. Cell-based studies offer extremely large sample sizes and do not require approval from regulatory agencies, resulting improved statistical power while decreasing both time and cost needed to conduct a study. Consequently they allow for the rapid study of drug response in a highly human relevant system for a fraction of the expense of traditional methods. Moreover, these assays can be made tissue or disease specific by using cultures of the relevant cell type, further increasing *in vivo* relevancy. A more detailed discussion of the advantages and limitations of such *in vitro* assays has previously been reviewed [1,2]. An increasing number of success stories for such experiments are emerging [3-9], but the statistical methodologies applied in such experiments have not been examined in detail.

**Figure 1:**
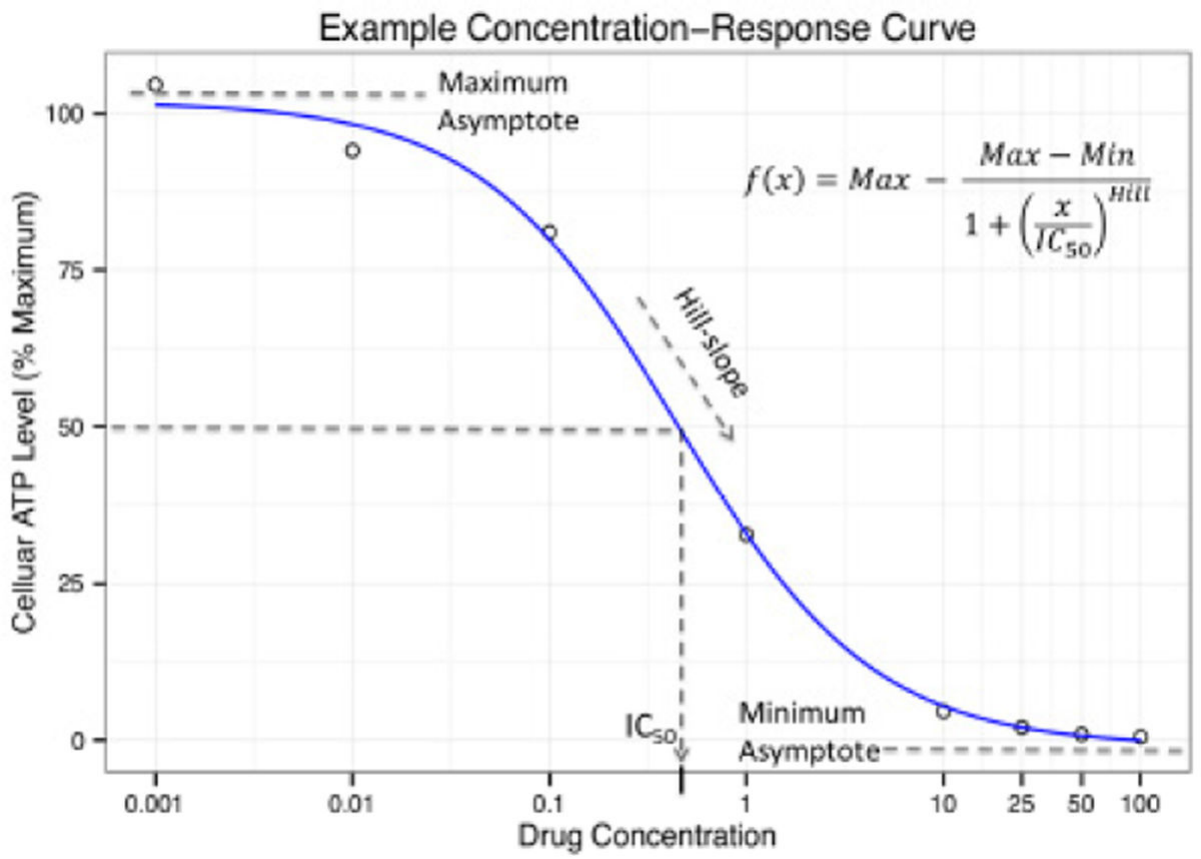
An example concentration-response curve with the 4 parameters (Maximum, Minimum, IC_50_, and hill-slope) of the hill-slope model labeled. The equation is displayed in the upper-right corner.

Cell-based studies allow for the examination of drug response at greater resolutions by measuring cellular response across a spectrum of concentrations rather than limited set of concentrations afforded by traditional studies. These types of *dose-response* or *concentration-response* studies measure some indication of cellular health or response such as total ATP, cell viability/morphology, or transcript expression levels as a function of increasing drug concentration. These data points are then fit to a statistical model, usually some form of a 4-parameter logistic curve (sometimes referred to as the *hill equation*), to produce a dose-response curve. Figure 1 shows an example of anannotated concentration-response curve. The curve is usually summarized by a single parameter such as the curve’s inflection point (e.g. the EC/IC_50_) [10] or the slope of the curve (called the *hill-slope*) [11]. Perhaps the most widely used summary in pharmacogenomics cell line experiments is the IC_50_, which represents the concentration where the response achieves 50% of maximal activity [3-9]. This notion of IC_50_ can be generalized further such that IC_X_ is the concentration at which the response is X% between minimal and maximal activity. IC_50_s (and their IC_X_ cousins) have been widely used in areas such as toxicology, pharmacology/pharmacogenomics, and industrial drug development [10]. Its popularity derives from the fact that it is a concise and interpretable summary of a drug’s activity, which conveys an indication of the drug’s *potency*. In association studies, this value is treated as a quantitative trait and standard QTL methods are then applied to link genotype to this derived phenotype [12].

However, traditional analytic and statistical methods are often illequipped to analyze this type of data and subsequent inference based on the IC_50_ poses many challenges. First, the appropriateness of the hillslope model from which the IC_50_ is derived is often unchecked, which may have large implications on the resulting conclusions [12]. This model is based on ligand-macromolecule binding dynamics [13], which may be an appropriate model in some instances, but inappropriate in others. Assuming this model is a correct description of the underlying biology, accurate calculation of the IC_50_ may still proves problematic. Estimating the IC_50_ using this model is highly sensitive to observing the full dose-response curve in the tested concentration range. If either the minimum or maximum asymptote of this curve is not observed it can have a very large impacton the estimated IC_50_ which will have a correspondingly large impact on the biological conclusions. Due to the non-linearity of this model even the “well-behaved” responses may result in unstable IC_50_ estimates. Two analysts may reach different IC_50_ values because they used different software packages or because they usedthe same software with different configuration settings. Such differences may cause the software to fail to produce a solution at all or may produce very different IC_50_ estimates, with no clear procedure for determining the correct value [14].

**Figure 2:**
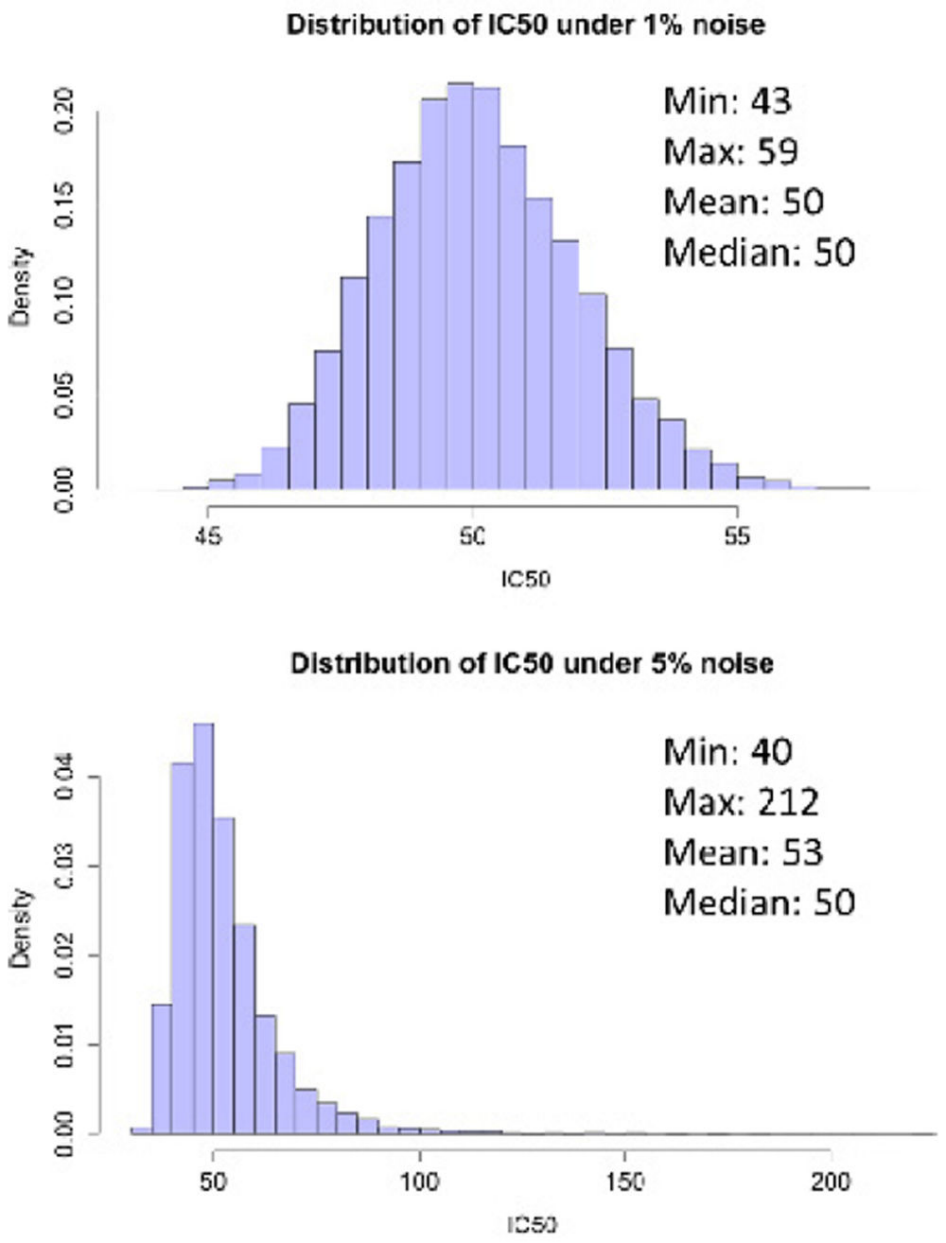
Distribution of the IC_50_ under two levels of noise. Note the wide range of estimated IC_50_s, especially under the slightly higher 5% noise model with many estimates being 2-3x larger the true value of 50 μM. Note also that the second histogram is no longer symmetric, implying that these IC_50_s are not normally distributed.

For example, assume a study measures total ATP across 8 concentrations using five technical replicates. Some summary statistic of these replicates, such as the mean or median ATP level at each concentration may then be fit to the hill-slope model to obtain an IC_50_ value. The amount of uncertainty introduced from sampling the response at each concentration can have a considerable impact on the estimated IC_50_. To highlight this issue and give some sense of how it can affect the IC_50_, we performed a small simulation experiment. For a concentration set of {0.0001,0.001,0.01,0.1,1,10,50,100} μM, we simulated 5 technical replicates for 2 levels of noise. Each technical replicate was generated from the hill-slope model plus a small amount of random noise with a true IC_50_ value of 50 μM. The noise was a random value of +/- X% of the true response value, where the values for X we tested were 1% and 5%. This is a ‘heteroskedastic’ noise model and is consistent with our experience the amount of variation in a response is proportional the size of the response itself. We then took mean of the 5 technical replicates and fit a curve using the *nls()* function in the R statistical language [15] to this mean response and recorded the estimated IC_50_. Note that we supplied the algorithm with true parameter values as starting values so as to minimize the amount of IC_50_ variation coming from the fitting process. We repeated this process 10,000 times for both levels of noise. Figure 2 shows histograms for the 1% and 5% noise level models. Even in the presence of a small amount of noise (1%), the IC_50_ estimates span a range of 40-70 μM and inspection of the estimated confidence interval for one such response yields a similar estimated range of variability. The situation for higher noise model is much worse with the IC_50_ estimate ranging from 40-212 μM, which was outside of the tested concentration range. This amount of uncertainty results in an IC_50_ measure that is not very useful in practice because it is statistically indistinguishable from a potentially wide range of other IC_50_ values. In the context of association studies, this could be of great harm. Imagine that there are two populations where one population has an *estimated* IC_50_ that is 2-3x that of the reference population, indicating that this population may be highly tolerant to the drug under study. It would be of great interest to locate any genetic loci that may be involved in this process. However, as the Figure 2 implies, these two populations may in fact have the same tolerance for the drug, but the noise introduced through sampling and estimating the IC_50_ has obfuscated our ability to see this, resulting in wasted time and effort looking for the underlying causes of a phantom difference.

This leads to yet another issue with IC_50_ based inference, namely that once all of the proper variation is accounted for, IC_50_s may show little meaningful variation in the statistical sense. This may result in two compounds, which by other measures would be considered to have different activity, to fail to be declared distinct, because their IC_50_s are not statistically separable. Statistical models are built to explain variation, but in the absence of meaningful variation, they will be unable to detect any genetic signal that may be present. This will be of increasing importance if pharmacogenomics, and genomics more generally, is to unravel complex traits that do not have large, single gene effects.

A somewhat larger point worthy of consideration is just how relevant an IC_50_, even one estimated with highprecision, is to the underlying scientific question. Statistical methods are only valid to the extent to which they mapback to the research question being asked. Even in the absence of all the issues discussed so far with IC_50_ based inference, it may be that a “true” statistically significant difference is not very meaningful from a biological perspective. Why might we assume *a priori* that *this* parameter from *this* model is the best representation of a compound’s activity? In this sense, it is not clear that IC_50_s are always a relevant measure or summary of a compound’s activity, if potency is not a meaningful proxy for the latent biological difference. If the IC_50_ is a poor proxy, then methods that take the full dose-response into consideration should be considered.

With both the promise of *in vitro* studies and the analytic challenges they present in mind, we hope to draw attention to some of the issues that must be addressed to maximize the utility of these types of assays. There are alternatives to the IC_50_ based significance testing approach that have been and continue to be developed. The area under the curve (AUC) statistic computes the area between the dose-response curve and the x-axis and is a global measure of compound’s activity [12]. This type of summary is potentially more robust than an interpolated parameter such as an IC_50_. Determination of statistical difference between two compound’s AUC relies on a permutation testing based procedure and may be very computationally expensive for large datasets. However, since permutation testing can be readily parallelized, the availability of computing clusters can reduce the time needed for this type of analysis. Multivariate ANOVA Genome-Wide Association Software (MAGWAS) [16] was shown to be a very attractive approach with many desirable properties including high statistical power and computational efficiency. However, MAGWAS is sensitive to changes that occur only at one concentration, which may not be desirable in some instances. Both AUC and MAGWAS incorporate the full dose response curve into the association tested.

Each of these concerns is only exaggerated by the increasingly high throughput nature of these experiments. As robotics has enabled rapid, high-throughput phenotyping for such experiments, investigators are now able to readily assay dozens or even hundreds of chemicals across hundreds of cell lines for dose response [17]. This makes it less likely that all assumptions are met or checked across such large numbers of results. This magnifies the importance of considered statistical approaches that minimize the impact of violations from these assumptions.

It is our hope that this discussion can help further the continued consideration on best practices for *in vitro* association studies. While we acknowledge that that IC_50_s can be of great utility when used properly and in the correct context, we hope to raise awareness of potential issues with these approaches and highlight alternatives that could further our understanding of gene based drug response.
